# Naturally Occurring Genetic Variants in the Oxytocin Receptor Alter Receptor Signaling Profiles

**DOI:** 10.1021/acsptsci.1c00095

**Published:** 2021-09-08

**Authors:** Manasi Malik, Michael D. Ward, Yingye Fang, Justin R. Porter, Maxwell I. Zimmerman, Thomas Koelblen, Michelle Roh, Antonina I. Frolova, Thomas P. Burris, Gregory R. Bowman, Princess I. Imoukhuede, Sarah K. England

**Affiliations:** † Center for Reproductive Health Sciences, Department of Obstetrics and Gynecology, Washington University School of Medicine in St. Louis, St. Louis, Missouri 63110, United States; ‡ Department of Biochemistry and Molecular Biophysics, Washington University School of Medicine in St. Louis, St. Louis, Missouri 63110, United States; § Department of Biomedical Engineering, McKelvey School of Engineering, Washington University in St. Louis, St. Louis, Missouri 63130, United States; ∥ Center for Clinical Pharmacology, Washington University School of Medicine in St. Louis and University of Health Sciences and Pharmacy in St. Louis, St. Louis, Missouri 63110, United States

**Keywords:** oxytocin receptor, molecular dynamics, β-arrestin, pharmacogenetics, variants of unknown significance, precision medicine

## Abstract

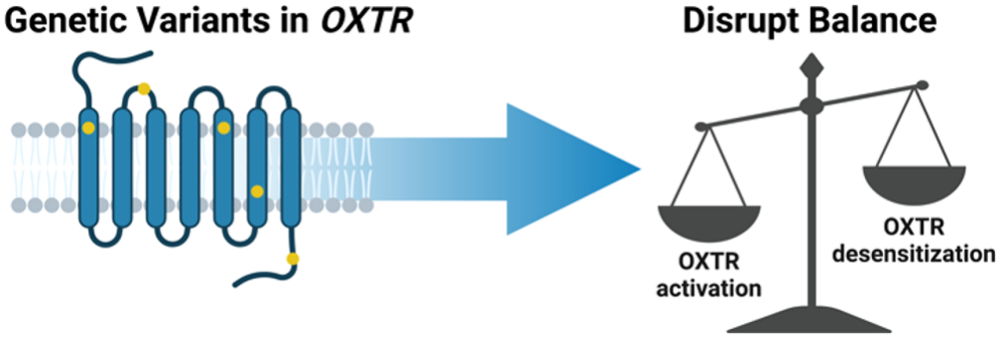

The hormone oxytocin is commonly administered during childbirth to initiate and strengthen uterine contractions and prevent postpartum hemorrhage. However, patients have wide variation in the oxytocin dose required for a clinical response. To begin to uncover the mechanisms underlying this variability, we screened the 11 most prevalent missense genetic variants in the oxytocin receptor (*OXTR*) gene. We found that five variants, V45L, P108A, L206V, V281M, and E339K, significantly altered oxytocin-induced Ca^2+^ signaling or β-arrestin recruitment and proceeded to assess the effects of these variants on OXTR trafficking to the cell membrane, desensitization, and internalization. The variants P108A and L206V increased OXTR localization to the cell membrane, whereas V281M and E339K caused OXTR to be retained inside the cell. We examined how the variants altered the balance between OXTR activation and desensitization, which is critical for appropriate oxytocin dosing. The E339K variant impaired OXTR activation, internalization, and desensitization to roughly equal extents. In contrast, V281M decreased OXTR activation but had no effect on internalization and desensitization. V45L and P108A did not alter OXTR activation but did impair β-arrestin recruitment, internalization, and desensitization. Molecular dynamics simulations predicted that V45L and P108A prevent extension of the first intracellular loop of OXTR, thus inhibiting β-arrestin binding. Overall, our data suggest mechanisms by which *OXTR* genetic variants could alter clinical response to oxytocin.

A synthetic form of the hormone oxytocin is administered to a large portion of pregnant patients in the United States to induce or augment labor^1^ and to nearly all patients who deliver to prevent postpartum hemorrhage.^2^ Oxytocin response varies widely between individuals.^3^ For labor induction and augmentation, maximal oxytocin infusion rates range from 2 milliunits/min (the starting rate specified in low-dose protocols) to 40 milliunits/min (the maximal infusion rate recommended by many providers).^3^ The duration of oxytocin infusion required before delivery also varies by 50 h or more, contributing to wide variations in the total oxytocin dose received by patients.^4^ Patients who receive high oxytocin doses are at increased risk for uterine hyperstimulation and rupture^5^ and postpartum hemorrhage secondary to uterine atony.^6−8^ In contrast, patients who receive insufficient oxytocin doses may require Cesarean delivery, which puts them at risk for surgical complications.^9^ To avoid these adverse events, clinicians have sought to identify individual factors that predict oxytocin dose requirement and thus enable personalized dosing of oxytocin.

The oxytocin receptor (OXTR) is a member of the G protein coupled receptor (GPCR) family. To bind to oxytocin, OXTR must first traffic to the myometrial smooth muscle cell surface. Upon oxytocin binding, OXTR activates Gq, leading to Ca^2+^ release from intracellular stores, which promotes myometrial smooth muscle contraction.^10^ OXTR signaling through Gq is counteracted by coupling to β-arrestin, which mediates desensitization and internalization of OXTR from the cell surface.^11−14^ OXTR desensitization after oxytocin exposure may impair myometrial contractions, leading to adverse events including uterine atony and postpartum hemorrhage.^6−8^

Several investigators have tested the hypothesis that variants in the *OXTR* gene affect the response to exogenous oxytocin. For example, Reinl et al. and Grotegut et al. identified single nucleotide *OXTR* variants in patients who required high or low doses of oxytocin to induce labor, but these studies were not powered to detect significant associations.^15,16^ In an *ex vivo* study, one coding and one noncoding *OXTR* variant altered the oxytocin-induced contractions of uterine tissue strips isolated from pregnant individuals.^17^ Although exome sequencing studies have shown that missense variants in the *OXTR* gene are prevalent in the global human population,^18^ the functional effects of most of these variants have not been determined. However, prevalent missense variants in other GPCRs genes lead to aberrant drug responses.^19^ Here, we assessed the effects of genetic variants of unknown significance in *OXTR* on oxytocin response in cells.

## Methods

### Cell Culture

HEK293T cells were maintained in Dulbecco’s modified Eagle’s medium/Ham’s F12 medium without phenol red and supplemented with 10% fetal bovine serum and 25 μg/mL gentamicin. Cells were kept in a humidified cell culture incubator at 37 °C with 5% CO_2_.

### cDNA Constructs

The wild-type (WT) OXTR and P108A OXTR constructs in pcDNA3.1(+) vector were a kind gift from Dr. Jeffrey Murray (University of Iowa). Other missense single nucleotide variants were introduced by site-directed mutagenesis (Genewiz, South Plainfield, NJ). The WT OXTR sequence was identical to the coding region of the National Center for Biotechnology Information reference sequence NM_000916.3.

The β-arrestin-1-Rluc8 fusion construct in the vector pcDNA3.1(+) encoded β-arrestin-1 with a C-terminal linker SGGSTSA followed by Rluc8. The β-arrestin-2-Rluc8 fusion construct in the vector pcDNA3.1(+) encoded β-arrestin-2 with a C-terminal linker GGGSEF followed by Rluc8. The template cDNA clones for β-arrestin-1 (ARRB100002) and β-arrestin-2 (ARRB200001) were obtained from the cDNA Resource Center (Bloomsberg, PA, www.cdna.org). A plasmid containing the Rluc8 cDNA was a kind gift from Dr. Brian Finck (Washington University in St. Louis).

The OXTR-GFP10 fusion construct in the vector pcDNA 3.1(+) encoded OXTR with a C-terminal linker SGGKL followed by GFP10. A plasmid containing the GFP10 cDNA was a kind gift Dr. Céline Gales (INSERM, France).

The plasmid encoding OXTR-GFP was a gift from Christian Gruber (Addgene plasmid #67848; http://n2t.net/addgene:67848; RRID: Addgene_67848).^20^ Note that this plasmid includes the missense single nucleotide variant A218T, which was corrected before introducing the variants of interest. An N-terminal HA tag was added (linker GPT) to generate the HA-OXTR-GFP construct.

All plasmids were confirmed by bidirectional Sanger sequencing.

Oxytocin (Tocris Bioscience, Minneapolis, MN) stock solutions diluted to 500 μM in water were stored at −80 °C until just before use.

### Ca^2+^ Assays

HEK293T cells (2 × 10^4^) were plated in each well of 96-well black-walled, clear-bottom polystyrene microplates coated with poly-d-lysine. The following day, cells were transfected with a construct encoding WT or variant OXTR. Each variant was tested alongside WT controls on the same plate. For transfections, 50 ng of DNA and 0.5 μL of TransIT-293 reagent (Mirus Bio, Madison, WI) diluted in Opti-MEM reduced-serum media (Thermo Fisher Scientific, Waltham, MA) were added to each well. After 24 h, media was removed and replaced with 100 μL of Brilliant Calcium indicator solution (Ion Biosciences, San Marcos, TX), which was prepared by diluting Brilliant Calcium indicator, DrySolv, and TRS reagent in assay buffer. After incubation for 1 h, a Synergy2 plate reader (BioTek, Winooski, VT) was used to add 100 μL of oxytocin of the appropriate concentration and record the fluorescence intensity (excitation filter = 485/20 nm, emission filter = 528/20 nm) every 0.14 s for 20 s/well. Fluorescence increase (increase in intracellular Ca^2+^) was calculated as the average of fluorescence intensity readings from 10 to 20 s after oxytocin addition minus the minimum fluorescence intensity averaged over five points from 0 to 10 s.

For desensitization assays, transfected cells were pretreated with the indicated oxytocin concentrations for 30 min. Then, without washing out the pretreatment oxytocin, a Synergy 2 plate reader was used to add a challenge dose of 1 μM oxytocin and record response as above.

### Bioluminescence Resonance Energy Transfer (BRET) Assays

HEK293T cells (4 × 10^4^) were plated in each well of 96-well white-walled, clear-bottom polystyrene microplates coated with poly-d-lysine. The following day, cells were transfected with WT or variant OXTR-GFP10 and β-arrestin-1-Rluc8 or β-arrestin-2-Rluc8 at a ratio of 15:1 (w/w). For transfections, 50 ng of DNA and 0.5 μL of Lipofectamine 2000 reagent (Thermo Fisher Scientific), both diluted in Opti-MEM reduced-serum media, were added to each well. After 24 h, media was removed and replaced with 100 μL of Hank’s buffered salt solution (HBSS) supplemented with 20 mM HEPES. A Synergy2 plate reader was used to add 100 μL of assay buffer containing 10 μM coelenterazine 400a (Biotium, Fremont, CA) and the indicated concentrations of oxytocin to 10 wells at a time. Luminescence at 520 and 400 nm was read every 26 s for a total of 182 s. The BRET ratio was calculated as the average ratios of emission at 520 nm/400 nm at the five time points from 78 to 182 s. WT controls were tested on each plate in parallel with variants.

### Quantitative Flow Cytometry

HEK293T cells (1 × 10^6^) were plated in T25 flasks and transfected the next day with HA-OXTR-GFP, OXTR-GFP, or HA-OXTR. Cells were transfected with 300 ng of plasmid DNA and 4 uL of TransIT-LT1 reagent (Mirus Bio). Cells were detached 24 h later with CellStripper (Corning) and collected by centrifugation. To measure receptor internalization, cells were incubated with the indicated concentration of oxytocin for 30 min before and during detachment. Cells were incubated with an empirically determined saturating concentration (8–16 μg/mL) of phycoerythrin (PE)-conjugated anti-HA antibody (901518, Biolegend, San Diego, CA) in staining buffer (0.5% BSA and 0.1% sodium azide in Ca^2+^/Mg^2+^-free PBS) on ice for 40 min and then washed twice in staining buffer before flow cytometry to quantify cell surface OXTR. For quantification of total OXTR, the PE-labeled living cells were fixed with 2% paraformaldehyde and permeabilized with 0.5% Tween20 in PBS. Cells were washed with 0.1% Tween 20 in PBS, incubated with 16 μg/mL PE anti-HA antibody for 40 min at room temperature, and washed twice before flow cytometry.

Flow cytometry was performed on a CytoFLEX flow cytometer (Beckman Coulter, Indianapolis, IN). Three technical replicates were performed for each experimental condition, and data from 5000 transfected cells were collected from each replicate. Three independent trials were performed. SYTOX Blue (Thermo Fisher Scientific) was used to exclude dead cells where appropriate. PE Quantibrite beads (BD Biosciences) were used for calibration. Flow cytometry gating was performed as follows: (1) forward and side scatter were used to exclude debris; (2) forward scatter width vs height was used to exclude doublets; (3) SYTOX blue staining was used to identify dead cells; (4) GFP fluorescence was used to gate transfected cells (GFP+ population). The GFP+ threshold was determined relative to the GFP signal in the GFP-negative control (cells transfected with HA-OXTR).

The number of receptors on transfected cells was calculated from the geometric mean of PE fluorescence intensity calibrated to PE standards as previously described.^21^ Values from nonspecific binding of PE-HA antibody to HA-negative cells (cells transfected with OXTR-GFP) were subtracted from all samples.

### Data Processing for Ca^2+^, BRET, Desensitization, and Internalization Assays

For Ca^2+^ and BRET assays, responses were normalized by subtracting the average basal response from all samples and then dividing by the average WT response at the highest oxytocin concentration for each trial. For desensitization and internalization experiments, responses were normalized by dividing values from all samples by the average response from the corresponding nonpretreated sample(s). Normalization was performed separately for each replicate experiment.

Nonlinear regression with least-squares fitting was used to generate dose–response curves and calculate *E*_max_, EC_50_, and IC_50_ values (GraphPad Prism 8). The three-parameter regression method, which was used to fit the BRET data and internalization data, used the model: *Y* = Bottom + (Top – Bottom)/(1 + 10^(log(EC_50_) or IC_50_–*X*)^). The four-parameter regression method, which was used to fit the Ca^2+^ activation and desensitization data, used the equation *Y* = Bottom + (Top – Bottom)/(1 + 10^((log(EC_50_) or IC_50_–*X*)×HillSlope))^. In these models, *Y* = response, *X* = log(oxytocin concentration), and no constraints were placed on any values. Buffer controls were assigned a nominal concentration value of 10^–9^ M for BRET assays or 10^–12^ M for all other assays.

All experiments were performed in triplicate, with WT controls tested alongside each variant on the same plate to control for day-to-day variation in assay response. Average values from three biological replicates were used to construct dose–response curves for each variant and the matched WT controls, which were compared by performing nested extra sum-of-squares F tests. F statistics were calculated and *P*-values were determined as previously described.^22,23^*P*-values shown reflect comparisons of log EC_50_ values or Top values (see equations above), as indicated.

### Molecular Dynamics Simulations

The initial homology model of WT OXTR was provided by the I-TASSER GPCR homology model database.^24^ This model was then prepared for simulation by the CHARMM-GUI membrane protein input generator.^25−28^ Mutations (e.g., V281M) and palmitate lipid tails on C346 and C347 were introduced by the CHARMM-GUI PDB manipulator.^29^ All proteins were simulated in 0.15 M KCl (111 K^+^ ions and 92 Cl^–^ ions) in a rectangular box of size 99.5 × 99.5 × 171.2 Å with a membrane consisting of 121 (upper leaflet) or 120 (lower leaflet) POPC molecules and 12 cholesterol molecules (upper and lower leaflet). All systems contained ∼100 000 TIP3P^30^ water molecules. Systems were minimized in the default manner supplied by CHARMM-GUI. Briefly, using the CHARMM36m force field,^31^ each system’s energy was minimized by using gradient descent, then simulated NVT with progressively weaker and fewer restraints on positions of atoms and membrane components.

Production runs were performed in GROMACS.^32^ Hydrogen bonds were constrained with the LINCS algorithm.^33^ Cutoffs of 1.2 nm were used for the neighbor list, Coulomb interactions, and van der Waals interactions. The force-switch modifier was used to smoothly switch forces from van der Waals interactions to zero between 1.0 and 1.2 nm. The Verlet cutoff scheme was used for the neighbor list. The Nosé–Hoover thermostat was used to hold the temperature at 300 K.^34^ The semi-isotropic Parrinello–Rahman barostat was used to maintain constant pressure of 1 bar as is standard in protein–membrane simulations.^35^ Conformations were stored every 20 ps.

The FAST algorithm^36,37^ was used to enhance conformational sampling for each OXTR sequence (WT, P108A, V281M, and V45L). Five FAST simulation rounds were conducted with 10 simulations per round. Each simulation was 50 ns in length (2.5 μs aggregate simulation). To explore away from the starting structure, the FAST ranking function favored restarting simulations from states that had the fewest number of preserved native contacts. Additionally, a similarity penalty was added to the ranking to promote conformational diversity in starting structures, as described previously.^38^

### DiffNet Analysis

DiffNets can perform dimensionality reduction in a way that highlights biochemically relevant differences between data sets.^39^ Two DiffNets were independently trained to learn about impairment of β-arrestin and Gq signaling. All DiffNet training and analysis was conducted under the assumption that the regions of Gq and β-arrestin binding were most likely to contain differences that explained impaired Gq or β-arrestin signaling. Therefore, the DiffNet analysis only considered atoms in the binding region (as shown in Figure S1). All simulation data (2.5 μs per variant) was converted to DiffNet input as described previously.^39^ Briefly, XYZ atom coordinates from simulations were mean-shifted to zero and then multiplied by the inverse of the square root of a covariance matrix, which was calculated from simulations. To learn about β-arrestin impairment, a DiffNet was trained to classify all structures from V45L and P108A as β-arrestin impaired (i.e., initial labels of one) and WT and V281M simulations as normal (i.e., initial labels of zero). To learn about Gq impairment, a DiffNet was trained to classify structures from V281M simulations as potentially Gq impaired and WT, V45L, and P108A simulations as normal. In both cases, the labels were iteratively updated in a self-supervised manner described previously^39^ in which expectation maximization bounds of [0.1–0.4] were chosen for normal variants and [0.6–0.9] for impaired variants. Both training sessions used 10 latent variables, 10 training epochs in which the data were subsampled by a factor of 10 in each epoch, a batch size of 32, and a learning rate of 0.0001.

### Markov State Model Construction and Analysis

A Markov State Model (MSM) is a statistical framework for analyzing molecular dynamics simulations and provides a network representation of a free energy landscape.^40−42^ To quantify differences between variants, several measurements were made that relied on MSMs, each built with 2.5 μs of simulation data for each variant. All MSMs were constructed with Enspara,^43^ a python library for clustering and building MSMs from molecular simulation data. In this work, Enspara was used to cluster OXTR structures, count transitions between clusters, and derive equilibrium probabilities of structural states explored during simulation. A separate MSM was built for each variant, using the same methodology for each variant. Namely, simulation frames were converted from XYZ atom coordinates to a vector containing a value indicating the amount of solvent-accessible surface area (SASA) of each residue side chain (i.e., the data was SASA featurized). SASA calculations were computed by using the Shrake–Rupley algorithm^44^ (with a solvent probe radius of 0.28 nm) as implemented in the python package MDTraj.^45^ SASA featurization was used for subsequent clustering because, unlike other clustering schemes (e.g., RMSD-based), SASA emphasizes the conformational changes of surface residues over internal residues, which should be most useful for understanding signaling of a transmembrane receptor that has a surface for binding ligands. Next, the SASA-featurized data were clustered with a hybrid clustering algorithm. First, a k-centers algorithm^46^ was used to cluster the data into 1000 clusters. Next, three sweeps of k-medoids update steps were applied to refine the cluster centers to be in the densest regions of conformational space. Then, transition probability matrices were produced by counting transitions between states (i.e., clusters) using a 2 ns lag time, adding a prior count of  and row-normalizing, as described previously.^47^ Equilibrium populations were calculated as the eigenvector of the transition probability matrix with an eigenvalue of one. For the distance histograms in Figures 6 and 7, the distance for each cluster center (i.e., representative structure of the cluster) was calculated and the distance was weighted by the corresponding equilibrium population calculated with the MSM. Similar calculations performed with an MSM built on an RMSD-based clustering scheme produced similar results (Figure S2).

## Results

### Genetic Variation Occurs in Several Locations within OXTR

**Figure 1:**
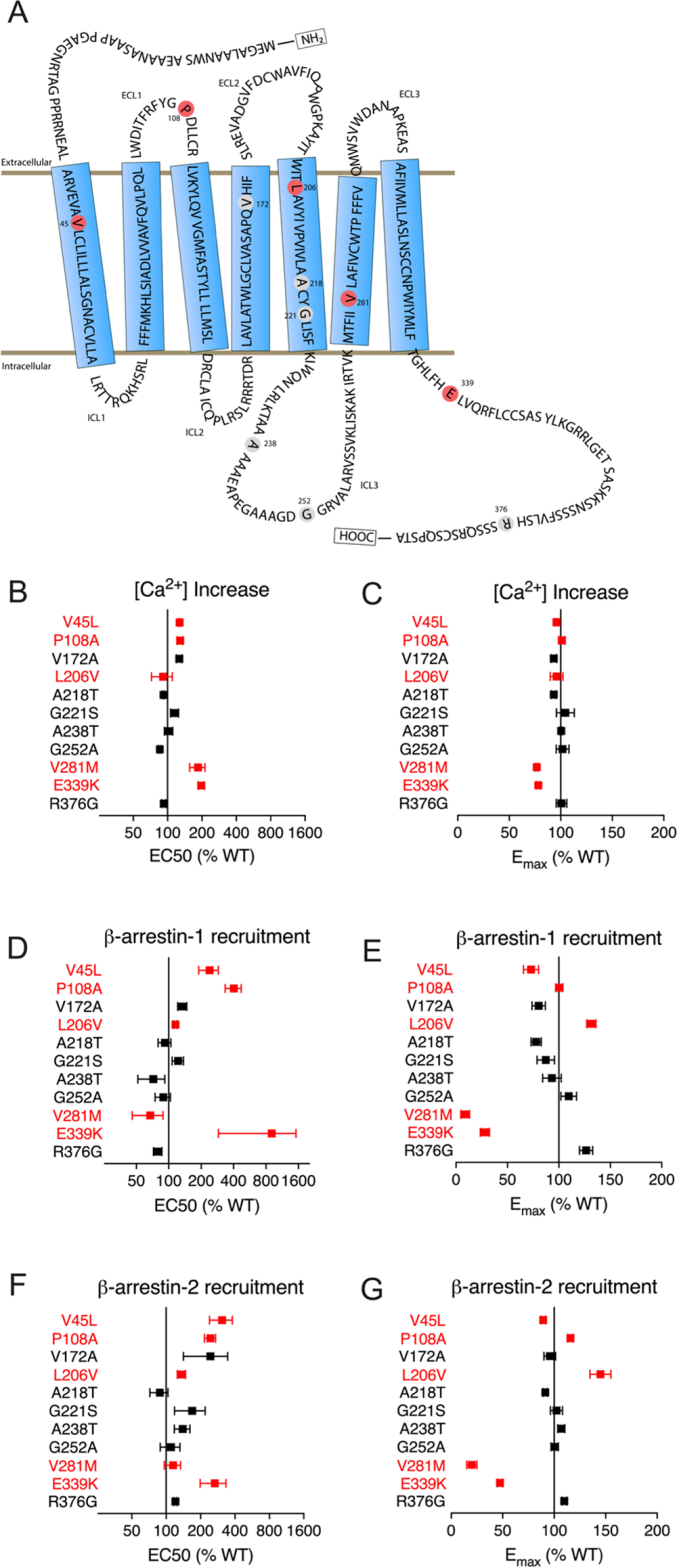
Screen identifies *OXTR* variants that alter oxytocin response in Ca^2+^assays and β-arrestin recruitment assays. (A) Variant residues within OXTR. ICL: intracellular loop. ECL: extracellular loop. (A–G) Plots show EC_50_ (B, D, F) and *E*_max_ (C, E, G) for dose–response curves for each variant, relative to WT value (100%). Variants shown in red were chosen for further study on the basis of large effect size and statistical significance (see Tables S1–S3). Error bars show standard error of the mean from *N* = 3 independent experiments with three to five technical replicates per experiment.

We searched the worldwide gnomAD v2.1 data set,^18^ which includes 141,456 exomes, to identify the most prevalent single nucleotide missense variants in *OXTR*. We identified 11 *OXTR* variants (Table 1) with allele counts greater than 50, indicating that they were detected in more than 50 heterozygous individuals.^18^ These variants affected residues in multiple domains, including six residues in transmembrane domains (TMs), one in the first extracellular loop (ECL1), two in the third intracellular loop (ICL3), and two in the C-terminal tail (Table 1, Figure 1A). The gnomAD cohort includes homozygotes for the four most common variants: A218T, A238T, V172A, and L206V. The most prevalent variant, A218T, was found in 27% of gnomAD participants; the 11th most prevalent variant, P108A, was found in 0.05% of participants.

**Table 1:** OXTR Variants for Study^a^

variant	location	allele count in gnomAD	affected (%)
A218T^5.56^	TM5	41 562	27.09
A238T	ICL3	5067	3.87
V172A^4.61^	TM4	1613	1.14
L206V^5.44^	TM5	551	0.39
E339K	C-terminus	308	0.22
G221S^5.59^	ICL3	215	0.15
G252A	ICL3	178	0.14
V281M^6.41^	TM6	107	0.08
V45L^1.38^	TM1	91	0.09
R376G	C-terminus	89	0.06
P108A	ECL1	74	0.05

a Affected (%): Percent of gnomAD participants with sequencing coverage at that locus who were homozygous or heterozygous for that variant. ECL: extracellular loop. ICL: intracellular loop. TM: transmembrane domain. Ballesteros–Weinstein numbering^61^ is shown for TM residues (superscripts).

### OXTR Missense Variants Alter Ca^2+^ Signaling and β-Arrestin Recruitment

We reasoned that the missense variants most likely to affect clinical oxytocin response would alter oxytocin-induced Ca^2+^ signaling, which is required for myometrial smooth muscle contraction, or recruitment of β-arrestin, which is thought to mediate OXTR desensitization.^13^ Therefore, to prioritize variants for further study, we transiently transfected plasmids encoding wild-type (WT) OXTR or the 11 variants into HEK293T cells and then performed high-throughput assays to measure effects on these pathways. First, to measure increases in intracellular Ca^2+^ in response to oxytocin, we used a fluorescent Ca^2+^ indicator dye. Second, to measure β-arrestin recruitment in response to oxytocin, we performed bioluminescence resonance energy transfer assays in HEK293T cells transfected with green fluorescent protein (GFP)-tagged OXTR and luciferase-tagged β-arrestin-1 or β-arrestin-2. V45L, P108A, L206V, V281M, and E339K had the largest statistically significant effects on EC_50_ or *E*_max_ in two or more assays and were therefore selected for further study (Figure 1, Tables S1–S3). V45L decreased the *E*_max_ for β-arrestin-1 recruitment and increased the EC_50_ for β-arrestin-2 recruitment (Figure S3). P108A increased the EC_50_ for β-arrestin-1 recruitment and increased both the EC_50_ and the *E*_max_ for β-arrestin-2 recruitment. L206V increased the *E*_max_ for β-arrestin-1 and β-arrestin-2 recruitment. V281M increased the EC_50_ for Ca^2+^ signaling and decreased the *E*_max_ for Ca^2+^ signaling and β-arrestin-2 recruitment. Finally, E339K increased the EC_50_ for Ca^2+^ signaling and decreased the *E*_max_ for Ca^2+^ signaling, β-arrestin-1 recruitment, and β-arrestin-2 recruitment (Figure 1).

### OXTR Variants Alter Cell Surface Localization

To quantify the effect of these five genetic variants on OXTR quantity and localization to the plasma membrane, we performed quantitative flow cytometry. A specific OXTR antibody is not commercially available, so we created a plasmid encoding the OXTR fusion protein HA-OXTR-GFP. We used GFP fluorescence to differentiate transfected from untransfected cells, and a phycoerythrin (PE) -conjugated anti-HA antibody to quantify the HA epitope on the extracellular N-terminus of OXTR. To quantify surface OXTRs, living cells were labeled by PE; to quantify total OXTRs throughout the cell, an additional PE-labeling step was performed after fixing and permeabilizing the PE-labeled living cells.

**Figure 2:**
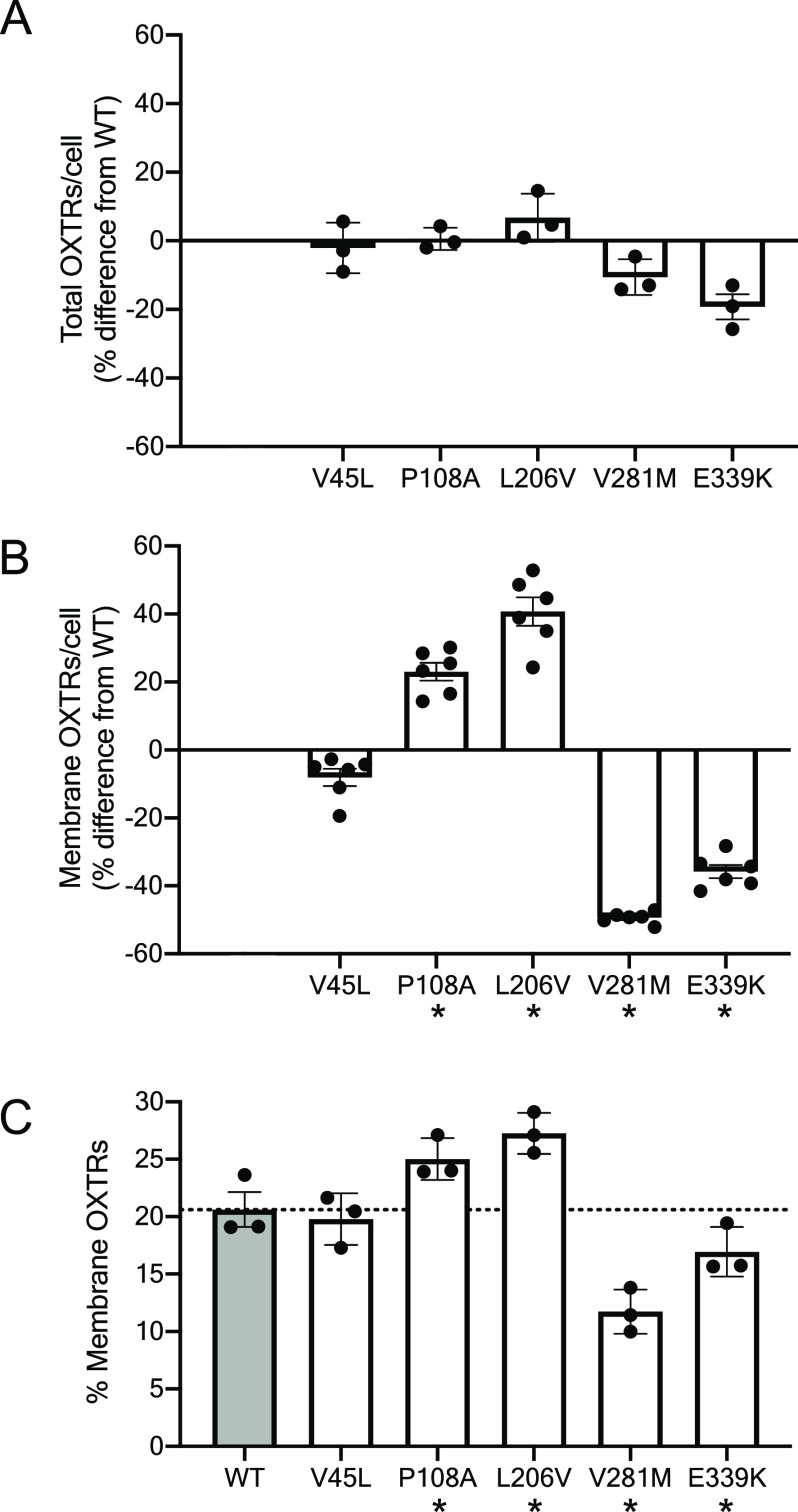
Genetic variants alter quantity of OXTR on the cell membrane. (A) Total number of OXTRs, (B) the number of OXTRs on the cell surface, and (C) the percentage of OXTRs on the cell surface in HEK293T cells transfected with plasmids encoding wild type (WT) and variant HA-OXTR-GFP. For (A) and (B), values for variants are shown as percent difference from the WT OXTR value. Error bars show standard error from *N* = 3–6 independent experiments with 15 000 cells across 3 technical replicates per experiment. Asterisk (*) indicates variant value differs from 0 with *P* < 0.01 in one-sample *t* test (B) or differs from WT with *P* < 0.05 in one-way repeated measures ANOVA with posthoc Dunnet multiple comparisons test (omnibus *P* = 0.0024) (C).

No variants had a statistically significant effect on the total number of OXTRs per cell after adjusting for multiple comparisons (*P* > 0.01 in one-sample *t* tests, Figure 2A). However, two variants (P108A and L206V) increased the number of cell surface OXTRs by 23 ± 3% and 41 ± 4%, respectively (*P* = 0.0003 and *P* = 0.0002, one sample *t* tests). Conversely, two variants (V281M and E339K) decreased the number of cell surface OXTRs by 49 ± 0.7% and 36 ± 2%, respectively (*P* < 0.0001, one-sample *t* tests, Figure 2B).

When we graphed cell surface OXTRs as a percentage of total OXTRs (Figure 2C), we found that 21 ± 2% of total WT OXTRs were localized to the plasma membrane. P108A and L206V increased OXTR surface localization to 25 ± 1% and 27 ± 1%, respectively (adjusted *P* = 0.03 for both). Conversely, V281M and E339K decreased OXTR surface localization to 12 ± 1% and 17 ± 1%, respectively (adjusted *P* = 0.01 for both).

### V45L, P108A, and E339K Impair OXTR Desensitization and Internalization

OXTR internalization and desensitization, mediated in part by β-arrestin recruitment, are thought to be responsible for some adverse effects associated with oxytocin exposure, including uterine atony and postpartum hemorrhage.^13^ Thus, to assess the potential clinical implication of variants, we aimed to define their effects on OXTR desensitization and internalization. As expected, for all five variants, relative differences in the number of cell surface receptors (Figure 2) corresponded to the differences seen in maximal β-arrestin recruitment assays (Figure 1E, G). For example, P108A and L206V had elevated *E*_max_ values for β-arrestin-2 recruitment and elevated membrane localization, whereas V281M and E339K had decreased *E*_max_ values for β-arrestin recruitment decreased membrane localization. In contrast, differences in the EC_50_ of β-arrestin recruitment did not correspond to changes in cell surface receptor number. For example, V45L increased the EC_50_ of β-arrestin-2 recruitment but had no effect on membrane localization, and P108A increased the EC_50_ of both β-arrestin-1 and β-arrestin-2 recruitment and increased membrane localization. We hypothesized that increased EC_50_ values would reflect functional deficits in OXTR desensitization and internalization.

**Figure 3:**
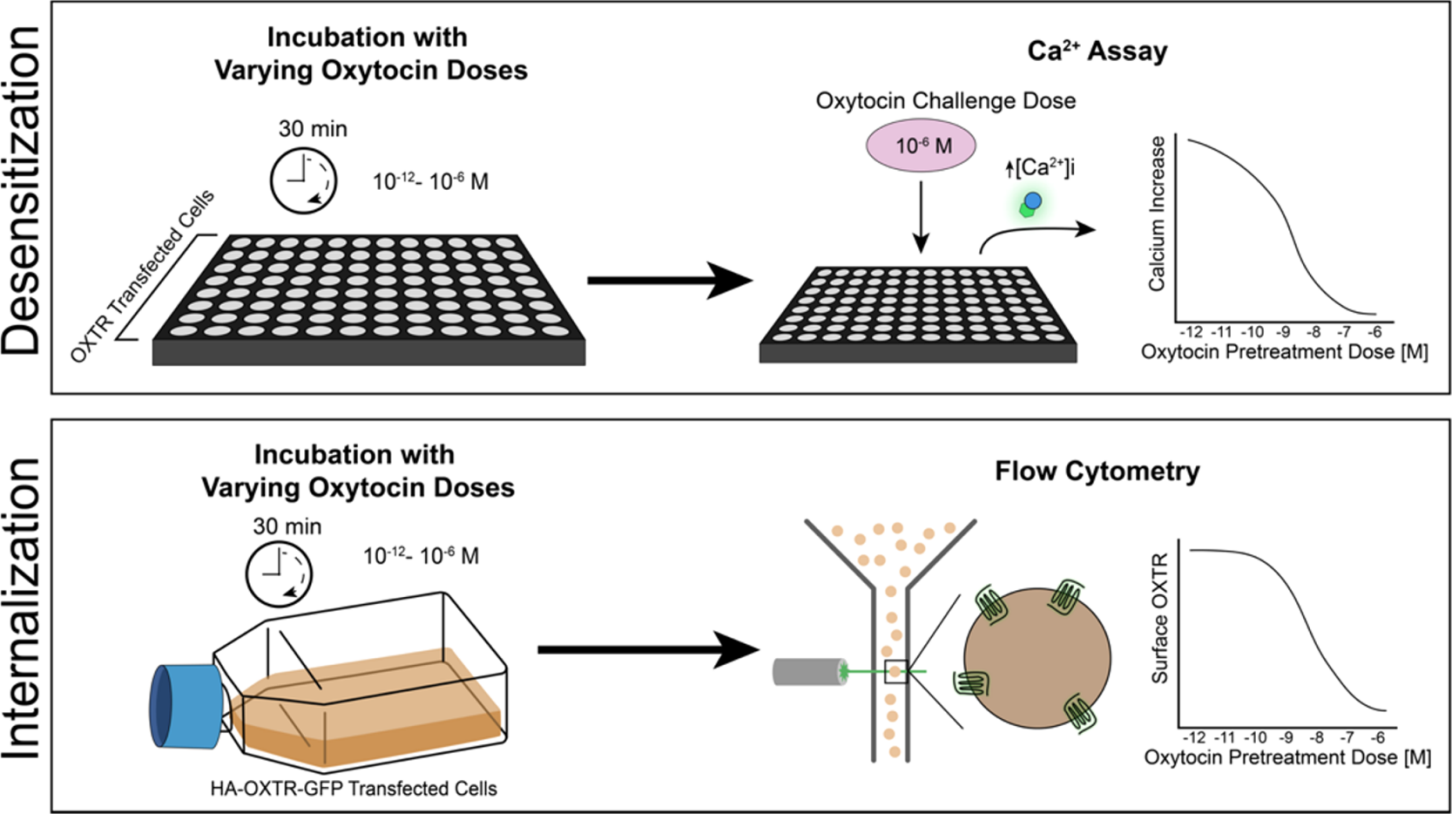
Method and data processing for desensitization and internalization assays. For desensitization assays, cells were incubated with indicated oxytocin doses for 30 min and then challenged with 1 μM oxytocin. The Ca^2+^ increase in response to 1 μM challenge is shown. For internalization assays, cells were incubated with indicated oxytocin doses for 30 min and then analyzed by quantitative flow cytometry to measure surface OXTR.

**Figure 4:**
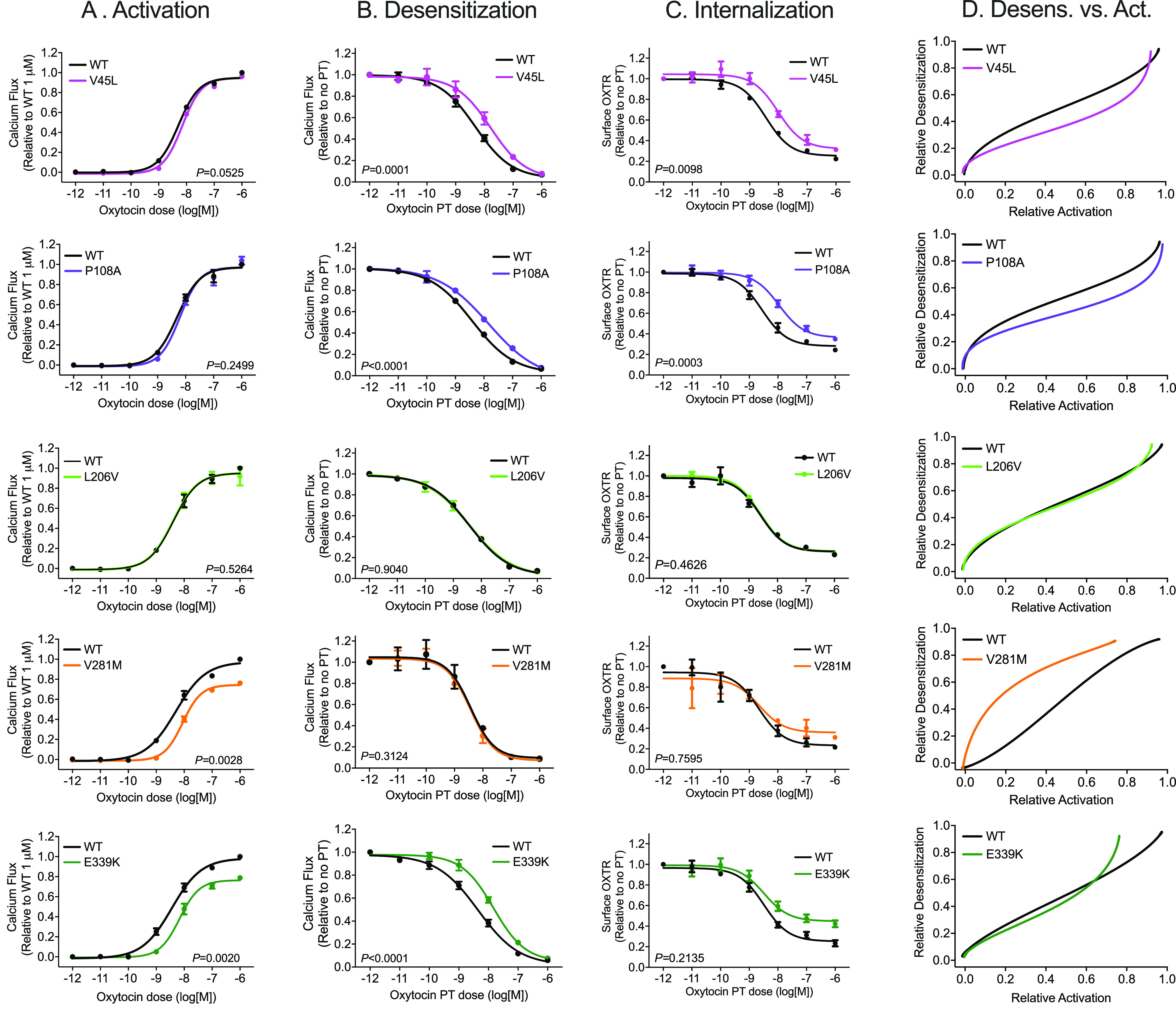
OXTR variants alter receptor activation, desensitization, and internalization. (A) Activation: increase in intracellular Ca^2+^ concentration in HEK293T cells transfected with wild type (WT) or variant OXTR and treated with oxytocin. Results are normalized to WT value at highest oxytocin concentration. (B) Desensitization: increase in intracellular Ca^2+^ concentration in cells treated with 1 μM oxytocin after pretreatment (PT) with the indicated oxytocin concentration. Results are normalized to response without PT. (C) Internalization of OXTR from the cell surface after PT with indicated oxytocin concentration. (D) Bias plot showing relative activation (*y* values from regression in (A)) and relative desensitization (regression of 1 – *y* from (B)). See also Figure S4. *P*-values for difference in log(EC_50_) or log(IC_50_) between WT and variant are shown (extra sum-of-squares F test, see also Tables S1 and S4). Error bars show standard error of the mean from *N* = 3 independent experiments.

To measure desensitization, we pretreated cells expressing WT OXTR or the five variants with varying concentrations of oxytocin for 30 min and then used Ca^2+^ indicator assays to measure the cellular response to a saturating concentration (1 μM) of oxytocin (Figure 3). To measure internalization, we incubated cells with varying concentrations of oxytocin for 30 min and then performed quantitative flow cytometry to measure surface OXTRs (Figure 3). We found that V281M and L206V had no effect on either receptor desensitization or internalization (*P* > 0.05, extra sum-of-squares F test). In contrast, V45L, P108A, and E339K caused a rightward shift in the dose–response curve and increased the IC_50_ for desensitization (*P* = 0.0001, *P* < 0.0001, and *P* < 0.0001, extra sum-of-squares F test, Figure 4B, Table S4). V45L and P108A caused a similar rightward shift in internalization assays (*P* = 0.0098 and *P* = 0.0003, extra sum-of-squares F test, Figure 4C, Table S4). Although E339K did not cause a statistically significant increase in EC_50_ for internalization (*P* > 0.05), it prevented maximal internalization, with 44% of E339K OXTRs versus 24% of WT OXTRs remaining on the cell surface (*P* = 0.0001, Figure 4C).

Three of the five variants investigated had differential effects on OXTR activation (oxytocin-induced Ca^2+^ signaling in Figure 4A), desensitization (Figure 4B), and internalization (Figure 4C). These variants altered the balance between OXTR desensitization and activation at any given dose of oxytocin (Figures 4D and S4). Of the three variants that impaired OXTR internalization and desensitization, only one, E339K, also altered potency and efficacy for OXTR activation, potentially due to decreased cell surface localization (Figure 2B). V281M had similar effects as E339K on OXTR cell surface localization and OXTR activation but had no effect on OXTR internalization or desensitization (Figure 4). In contrast, V45L and P108A impaired OXTR internalization and desensitization without altering OXTR activation (Figure 4).

### Variants that Reduce Desensitization and Internalization Alter OXTR Structural Conformations

**Figure 5:**
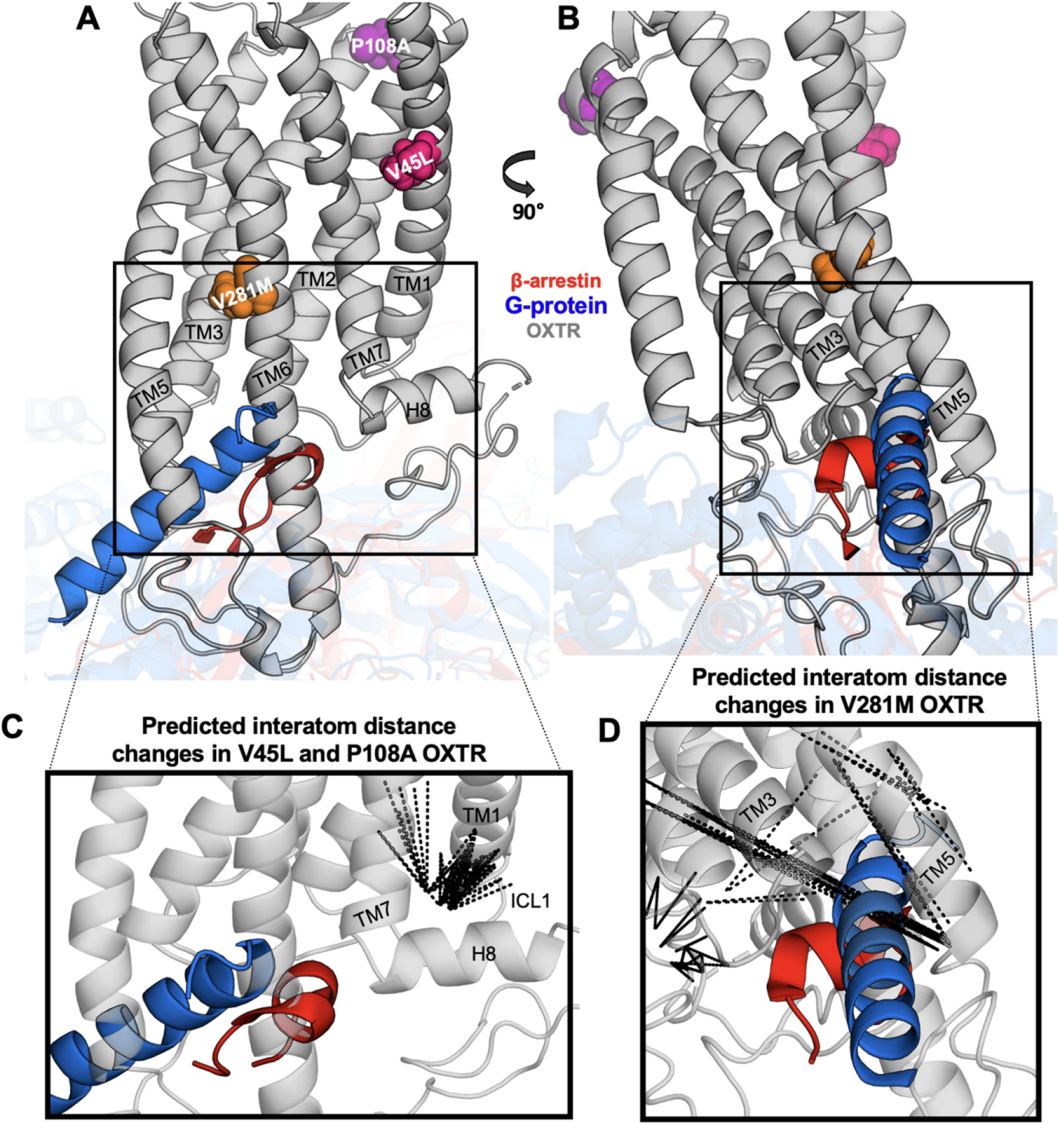
DiffNets identify distances associated with V45L, P108A, and V281M OXTR. (A, B) Homology model for OXTR showing the location of V45L, P108A, and V281M. Structures for β-arrestin-1 (red, PDB: 6pwc(48)) and Gαs (blue, PDB: 3sn6(49)) are superimposed on the OXTR structure. (C) Dotted lines show the 100 interatom distance changes most associated with DiffNet label (V45L and P108A vs WT and V281M). (D) Distance changes most associated with DiffNet label (V281M vs WT, V45L, P108A). TM: transmembrane domain. ICL1: intracellular loop 1. H8: helix 8.

In our *in vitro* assays, two variants (V45L and P108A) reduced β-arrestin recruitment, OXTR internalization, and OXTR desensitization compared to WT OXTR. Thus, three lines of evidence suggest that V45L and P108A decrease OXTR’s ability to activate β-arrestin. To define the structural basis of β-arrestin impairment, we used molecular dynamics simulations to computationally model the motions of all atoms in WT and variant OXTRs in solution over time (Figure 5A, B). We paired these simulations with the FAST algorithm (see Methods(36,37)) to enhance sampling of the conformational ensemble (i.e., the set of structural poses the receptor adopts) of each variant.

To identify the conformational changes most associated with β-arrestin impairment, we used DiffNets, deep-learning algorithms that are trained to identify biochemically relevant differences between multiple conformational ensembles (see Methods).^39^ We first trained a DiffNet to identify differences between conformational ensembles of the two β-arrestin-impaired OXTRs (V45L and P108A) and two OXTRs (WT and V281M) with normal desensitization and internalization. From this training, the DiffNet learned a label for each simulation frame (structural configuration) from zero to one that indicated the probability that it was associated with this classification. To interpret these labels, we calculated the correlation between interatom distances in the OXTR cytosolic region (71 289 possible distances, Figure S1) and changes in the DiffNet label. We then plotted the 100 distances that were most correlated with the DiffNet label (Figure 5C). This analysis showed clear enrichment in distances that cluster at the interface between transmembrane domain 1 (TM1) and the first intracellular loop (ICL1), indicating that changes in this region were associated with β-arrestin impairment.

### Conformational Changes in V45L and P108A OXTRs Disrupt Putative β-Arrestin Binding Sites

**Figure 6:**
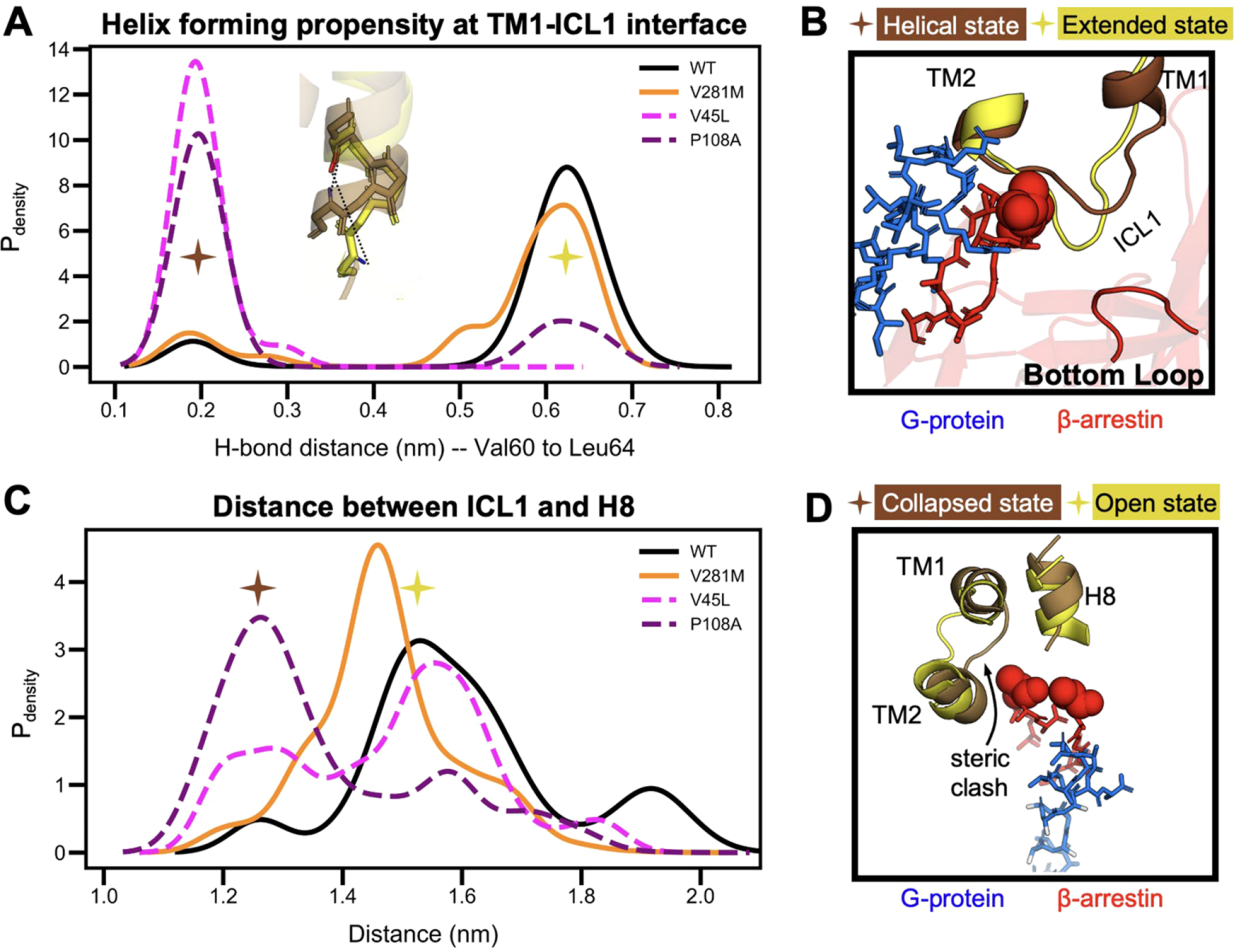
Potential mechanism for altered β-arrestin function in V45L and P108A OXTR. (A) Distribution of the probability-weighted density for the hydrogen bond distance between the most C-terminal TM1 helix *i*, *i* + 4 residue pair (Val^60^ and Leu^64^). β-Arrestin-impaired variants (V45L and P108A) have a high probability of having a tight helix, whereas OXTRs with normal desensitization and internalization (WT and V281M) are more likely to lack this hydrogen bond. (B) Representative structures from each peak in (A). The β-arrestin-1 “bottom loop” (red), which is involved in binding to ICL1, is closer to ICL1 when ICL1 is extended. (C) Distribution of the probability-weighted density for ICL1-H8 distances that each OXTR variant occupies. V45L and P108A have strong, left-shifted peaks indicating a collapse between ICL1 and H8. (D) Representative structures of ICL1-H8 at collapsed distances (brown) and open distances (yellow). In the collapsed position, there is a steric clash between ICL1 and β-arrestin. TM: transmembrane domain. ICL1: intracellular loop 1. H8: helix 8.

DiffNets identified locations associated with reduced β-arrestin function without any prior information about functional sites in OXTR. To determine whether the DifffNet predictions corresponded to functional locations, we used the simulation data to build Markov State Models. Markov State Models provide a discrete map of structural configurations and an equilibrium population value that corresponds to the proportion of time a protein spends in a given configuration.^40−42^ The DiffNet prediction implicated the TM1-ICL1 region in β-arrestin impairment, so we used Markov State Models to more closely examine this region. In this analysis, V45L and P108A introduced an additional helical turn at the C-terminus of TM1 that was not present in WT and V281M OXTR. Specifically, we found that the hydrogen bond between Val^60^ and Leu^64^ was shorter in V45L and P108A OXTR than in WT and V281M OXTR (0.2 vs 0.6 nm) (Figure 6A). Thus, β-arrestin-impaired OXTRs were predicted to have a shorter ICL1 than OXTRs with normal β-arrestin function.

This conformational change has important implications for β-arrestin binding. First, shortening ICL1 may prevent the interactions between ICL1 and the bottom loop of β-arrestin (Figure 6B) previously described by Yin et al.^48^ Second, shortening ICL1 reduces the distance between ICL1 and helix 8 (H8), causing a collapsed state (Figure 6C). When we superimposed bound structures of β-arrestin and G protein (from other GPCRs^48,49^) onto the OXTR homology model, the model predicted that this shortened distance created a steric clash between ICL1 and the β-arrestin finger loop, but not between ICL1 and the G protein (Figure 6D**)**. Taken together, our data suggest that the mechanism underlying reduced β-arrestin function was similar in V45L and P108A OXTR.

### Structural Conformations in V281M OXTR

Our results in Figure 4D indicated that the balance between OXTR activation and desensitization in V281M OXTR deviated significantly from WT, with greater relative desensitization for any given unit of activation. We observed the opposite deviation in V45L and P108A OXTR, both of which had less relative desensitization for any given unit of activation. To investigate the structural basis of this difference, we used a similar approach as above and trained a second DiffNet to identify differences between conformational ensembles of V281M OXTR and V45L, P108A, and WT OXTR. We plotted the 100 distances that were most correlated with the DiffNet label in Figure 5D. This analysis showed enrichment for distances between transmembrane domains 3 and 5 (TM3 and TM5), indicating that structural rearrangements in this region were associated with V281M.

**Figure 7:**
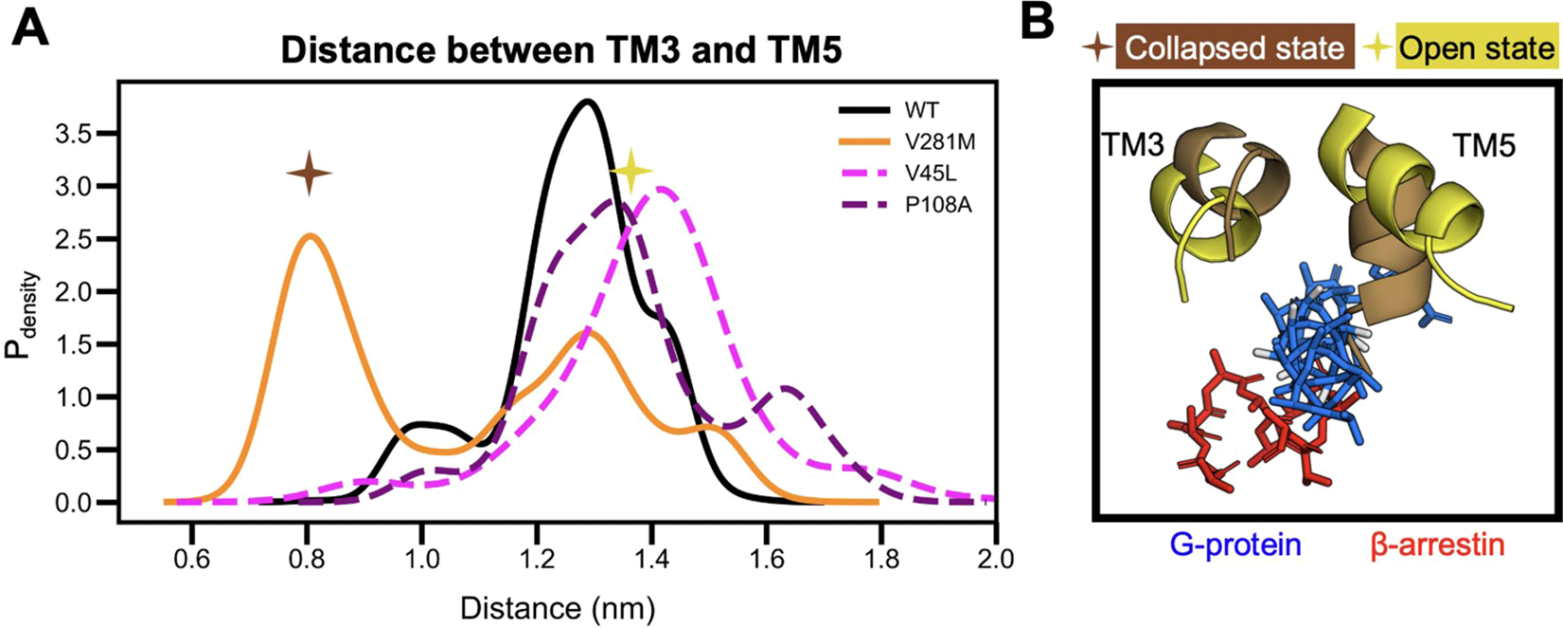
Conformational changes in V281M OXTR may reduce G protein binding. (A) Histogram showing a probability-weighted distribution of TM3-TM5 distances that each OXTR variant occupies. V281M OXTR is highly likely to adopt a collapsed state that would sterically hinder G protein binding. (B) Representative structures of TM3 and TM5 at collapsed distances (brown) and open distances (yellow). The collapsed position sterically clashes with the G protein (blue) but not β-arrestin (red). TM: transmembrane domain.

We then used Markov State Models to plot the probability that OXTR adopts a conformation with a given distance between TM3 and TM5. V281M OXTR was more likely to adopt conformations with a shorter distance between TM3 and TM5 than were WT, V45L, and P108A OXTR (0.8 nm versus 1.2–1.4 nm, Figure 7A). When we superimposed the bound β-arrestin and G protein structures, we saw that this collapsed state caused a steric clash with the G protein but not with β-arrestin (Figure 7B). This finding suggests that V281M disrupted the binding of Gq to OXTR without affecting β-arrestin recruitment.

## Discussion

Our data indicate that *OXTR* variants found in the global human population significantly altered OXTR function. Specifically, these variants altered oxytocin response by changing OXTR localization to the cell membrane, decreasing oxytocin-induced Ca^2+^ signaling, altering β-arrestin recruitment and signaling, or a combination of these effects. The variants P108A and L206V increased the percentage of OXTR on the cell membrane, whereas V281M and E339K caused OXTR to be retained inside the cell. V281M and E339K also decreased Ca^2+^ signaling. Three variants (V45L, P108A, and E339K) impaired OXTR desensitization and OXTR internalization upon exposure to oxytocin. Our molecular dynamics simulations predict that both V45L and P108A introduce an extra helical turn at the end of TM1, which may explain the impaired coupling to β-arrestin seen *in vitro*.

Our results from the V281M and E339K variants highlight the importance of efficient membrane trafficking for receptor function. These intracellularly retained variants were the only two variants studied that decreased oxytocin-induced Ca^2+^ signaling (Figure 1B, C). In contrast, P108A and L206V, which increased the number of OXTR on the cell surface, did not increase maximal Ca^2+^ signaling. This may be because Ca^2+^ signaling becomes saturated at a certain concentration of receptors per cell. Because Gq signaling amplifies through the signaling pathway that leads to Ca^2+^ mobilization, intracellular Ca^2+^ is not a one-to-one readout of Gq activation.^50^ A more direct measurement of Gq activation may show that maximal Gq activation correlates with surface OXTRs, but this may not translate directly to the activation of downstream pathways important for myometrial contractions.

Unlike maximal Ca^2+^ signaling, maximal recruitment of β-arrestin measured in the bioluminescence resonance energy transfer screen closely matched the number of OXTRs on the cell membrane. P108A and L206V, which increased cell surface OXTR, caused higher maximal recruitment (*E*_max_), whereas V281M and E339K, which decreased cell surface OXTR, caused lower maximal recruitment (Figure 1E, G). Changes in *E*_max_ in our bioluminescence resonance energy transfer assays seemed to reflect a change in the number of receptors available to recruit β-arrestin but did not always correspond to functional changes in receptor desensitization or internalization (Figure 4). For example, the L206V and V281M variants had the largest effects on *E*_max_ for β-arrestin recruitment but did not alter receptor desensitization or internalization. In contrast, increases in the EC_50_ for β-arrestin recruitment corresponded to right shifts in desensitization and internalization curves. Whereas OXTR desensitization and internalization can occur by several mechanisms, our results suggest that changes in β-arrestin recruitment EC_50_ translate to functional differences in desensitization and internalization.

To complement our *in vitro* assays, we used an *in silico* method to model the behavior of variant OXTRs. Our *in vitro* assays showed that V45L and P108A caused rightward shifts in the dose–response curves for β-arrestin recruitment, OXTR desensitization, and OXTR internalization but not oxytocin-induced Ca^2+^ signaling. We used the deep-learning approach DiffNets to identify structural changes that were common to V45L and P108A OXTRs but not present in OXTRs with normal internalization and desensitization. Importantly, the DiffNet required no input of information about OXTR/GPCR structure/function relationships to identify locations in OXTR that appear to be associated with β-arrestin binding. This discovery-based approach yielded predictions that correspond with our *in vitro* data as well as published work on the mechanism of β-arrestin binding in other GPCRs.^48^ The structural differences shown in Figure 6 suggest one mechanism by which OXTR can bind to and activate G proteins without activating β-arrestin. However, further work is necessary to validate these predictions and determine the mechanism of β-arrestin binding to OXTR. In the future, these findings may guide the design of biased agonists, as recently demonstrated by Suomuoviri et al. for the angiotensin II type 1 receptor.^51^ Novel uterotonics that mimic the effects of V45L and P108A may preferentially activate OXTR signaling through Gq with less β-arrestin activation, thus decreasing the risk of adverse effects associated with OXTR internalization and desensitization.

We used a similar approach to identify conformational changes associated with V281M, a variant that decreased OXTR activation (oxytocin-induced Ca^2+^ signaling) but had no effect on desensitization or internalization. Our Markov State Models predicted conformational changes in V281M OXTR consistent with steric hindrance of G protein binding (Figure 7). Importantly, these changes would not hinder binding of β-arrestin and thus present a possible mechanism by which V281M altered Ca^2+^ signaling without altering desensitization or internalization. However, the changes caused by V281M were also likely due, at least in part, to inefficient cell membrane localization of V281M OXTR (Figure 2). Therefore, further *in vitro* studies are necessary to determine whether V281M OXTR displays decreased binding to Gq and thus validate the predictions from our molecular dynamics simulations.

Our findings add to two previous *in vitro* studies examining human *OXTR* variants. First, Ma et al. showed that R376G, a variant associated with autism spectrum disorder, increased the rate of OXTR internalization and recycling to the cell surface after treatment with oxytocin.^52^ It is unclear whether the small changes in β-arrestin recruitment seen in our screening assays (Tables S2 and S3) explain the differences in OXTR internalization and recycling observed by Ma et al. Second, Kim et al. characterized three missense *OXTR* variants, including P108A, that they identified in patients who experienced premature labor.^53^ These authors reported that P108A decreased oxytocin binding but did not significantly affect Gq activation as measured by inositol phosphate production, which was consistent with our results. Furthermore, our findings show that P108A impaired OXTR desensitization, meaning that some OXTR Gq activation occurred unopposed. This could result in premature initiation of uterine contractions and thus explain an association between P108A and premature labor. Future studies are needed to determine whether P108A—and V45L, which we found to have similarly impaired desensitization and structural changes—predispose patients to preterm labor.

Understanding how genetic variants alter receptor function is an important step toward personalized drug dosing. Our functional annotation of the 11 most prevalent variants of unknown significance in *OXTR* helped us to prioritize the variants most likely to affect OXTR function for further study. These variants caused EC_50_ changes in the 2–4-fold range, consistent with effects caused by other naturally occurring GPCR variants linked to disease risk and drug response.^54−57^ Additionally, our data indicate that the two most prevalent missense variants, A218T and A238T, are unlikely to appreciably affect OXTR function.

Both activation and desensitization of the Ca^2+^ signaling pathway play an important role in determining clinical response to oxytocin. Currently, most oxytocin dosing protocols for labor induction call for providers to increase the oxytocin infusion rate at steady intervals, which compensates for a given amount of OXTR desensitization over time.^58^ Imbalance between these processes, also known as signaling bias, may therefore have clinical consequences, as shown in other GPCRs.^19,57,59^ In our study, we identified three variants that may cause signaling bias: (1) V45L and P108A impaired OXTR desensitization but not activation and (2) V281M decreased OXTR activation but not desensitization. However, further studies are necessary to determine whether these changes represent signaling bias between β-arrestin and Gq. Our data indicate that individuals who carry the V281M allele may be less responsive to oxytocin but still susceptible to the potential adverse effects that result from OXTR desensitization during labor (i.e., postpartum hemorrhage, uterine atony). These individuals may require higher doses of oxytocin to achieve labor induction and thus may have increased risk of these adverse events. Furthermore, oxytocin may be less effective in preventing postpartum hemorrhage in these individuals. In contrast, patients with V45L or P108A variants may be less susceptible to the adverse effects that result from OXTR desensitization but more susceptible to uterine hyperstimulation as a result of induction with oxytocin. Finally, patients with the E339K variant, which impairs OXTR activation and desensitization to roughly the same extent, may require higher oxytocin doses to achieve clinical effects.

Our studies indicate that individuals who carry the V45L, P108A, V281M, or E339K variants may benefit from personalized oxytocin dosing protocols or alternative methods of labor induction. P108A is found in 0.3% of the Finnish population, V281M is found in 0.7% of the Swedish population, and E339K is found in 1.5% of the Ashkenazi Jewish population.^18,60^ Further studies in these populations are necessary to determine the utility of genetic analyses in developing precision medicine approaches to oxytocin dosing.

## Supporting Information Available

Results from functional screen; log(IC_50_) for desensitization and internalization assays; additional bias plots for wild type and variant OXTRs; atoms included in DiffNets analysis (PDF)
