# Supplementary material for: Naturally Occurring Genetic Variants in the Oxytocin Receptor Alter Receptor Signaling Profiles
Source: ACS Pharmacol Transl Sci. 2021 Sep 8;4(5):1543–55. doi: 10.1021/acsptsci.1c00095 (PMC8506602; doi:10.1021/acsptsci.1c00095)
Supplement: Supplementary file 1 — pt1c00095_si_001.pdf [file pt1c00095_si_001.pdf]

Supporting Information

Naturally-occurring genetic variants in the oxytocin receptor alter receptor signaling profiles

Manasi Malik[1], Michael D. Ward[2], Yingye Fang[3], Justin R. Porter[2], Maxwell I. Zimmerman[2], Thomas Koelblen[4], Michelle Roh[1], Antonina I. Frolova[1], Thomas P. Burris[4], Gregory R. Bowman[2], Princess I. Imoukhuede[3], Sarah K. England*[1]

Affiliations:

[1] Center for Reproductive Health Sciences, Department of Obstetrics and Gynecology, Washington University School of Medicine in St. Louis, St. Louis, Missouri, USA
[2] Department of Biochemistry and Molecular Biophysics, Washington University School of Medicine in St. Louis, St. Louis, Missouri, USA
[3] Department of Biomedical Engineering, McKelvey School of Engineering, Washington University in St. Louis, Missouri, USA
[4] Center for Clinical Pharmacology, Washington University School of Medicine in St. Louis and University of Health Sciences and Pharmacy in St. Louis, St. Louis, Missouri, USA

*Correspondence: Sarah K. England (englands@wustl.edu)

Table of Contents

Table S1: Oxytocin response in Ca^{2+} assays for wild type (WT) and variant OXTRs....................2
Table S2: Oxytocin-induced β-arrestin-1 recruitment for wild type (WT) and variant OXTRs 3
Table S3: Oxytocin-induced β-arrestin-2 recruitment for wild type (WT) and variant OXTRs 4
Table S4: Log(IC50)s for desensitization and internalization curves for wild type (WT) and variant OXTR..5
Figure S1: Atoms included in DiffNets analysis 6
Figure S2: Comparison of equilibrium properties calculated from simulations using different clustering methods 7
Figure S3: Oxytocin-induced ß-arrestin recruitment to wild type (WT) and variant OXTRs.........8
Figure S4: Bias plots for wild type (WT) and variant OXTRs...9

Table S1: Oxytocin response in Ca^{2+} assays for wild type (WT) and variant OXTRs

Log(EC50)

	Variant log(EC50) (95% CI)	WT log(EC50) (95% CI)	Significance (P)
**V45L**	-8.165 (-8.237 to -8.099)	-8.266 (-8.349 to -8.165)	0.0525
**P108A**	-8.195 (-8.355 to -8.195)	-8.307 (-8.432 to -8.185)	0.2499
**V172A**	-8.295 (-8.444 to -8.161)	-8.396 (-8.531 to -8.265)	0.2879
**L206V**	-8.432 (-8.657 to -8.212)	-8.352 (-8.492 to -8.208)	0.5264
**A218T**	-8.435 (-8.503 to -8.368)	-8.399 (-8.483 to -8.316)	0.4842
**G221S**	-8.465 (-8.655 to -8.277)	-8.533 (-8.638 to -8.427)	0.5237
**A238T**	-8.533 (-8.679 to -8.386)	-8.543 (-8.681 to -8.403)	0.9157
**G252A**	-8.734 (-8.932 to -8.516)	-8.659 (-8.769 to -8.545)	0.5224
**V281M**	-8.054 (-8.115 to -7.989)	-8.301 (-8.449 to -8.136)	**0.0028**
**E339K**	-8.167 (-8.282 to -8.055)	-8.445 (-8.572 to -8.314)	**0.0020**
**R376G**	-8.699 (-8.900 to -8.480)	-8.650 (-8.763 to -8.532)	0.6732

E_{max}

	Variant E_{max} (95% CI)	WT E_{max} (95% CI)	Significance (*P*)
**V45L**	93 (89 to 96)	97 (93 to 101)	0.0783
**P108A**	98 (91 to 107)	96 (91 to 102)	0.7520
**V172A**	91 (86 to 97)	98 (92 to 103)	0.0868
**L206V**	93 (85 to 102)	98 (92 to 104)	0.0783
**A218T**	91 (89 to 94)	98 (95 to 102)	**0.0019**
**G221S**	103 (95 to 111)	98 (94 to 102)	0.2751
**A238T**	100 (94 to 106)	99 (94 to 105)	0.8697
**G252A**	101 (93 to 109)	99 (95 to 103)	0.7182
**V281M**	74 (72 to 77)	97 (91 to 105)	**<0.0001**
**E339K**	76 (72 to 81)	98 (94 to 104)	**<0.0001**
**R376G**	98 (91 to 107)	98 (94 to 103)	0.9194

Results shown are point estimates and 95% confidence intervals from dose-response curves generated from three replicate experiments. Variant point estimates are shown next to point estimate from the WT control on the same plate. Statistically significant changes in log(EC50) or E_{max} are shown in bold [(extra sum of squares F test, $P<0.0045$ ($\alpha=0.05$ with Bonferroni correction for 11 comparisons)].

Table S2: Oxytocin-induced β-arrestin-1 recruitment for wild type (WT) and variant OXTRs

Log(EC50)

	**Variant log(EC50) (95% CI)**	**WT log(EC50) (95% CI)**	**Significance (*P*)**
**V45L**	-6.842 (-7.095 to -6.587)	-7.232 (-7.419 to -7.038)	0.0139
**P108A**	-6.793 (-6.990 to -6.593)	-7.363 (-7.474 to -7.249)	**<0.0001**
**V172A**	-7.083 (-7.334 to -6.829)	-7.232 (-7.419 to -7.038)	0.3240
**L206V**	-7.416 (-7.637 to -7.187)	-7.456 (-7.598 to -7.309)	0.7793
**A218T**	-7.411 (-7.665 to -7.148)	-7.363 (-7.474 to -7.249)	0.7072
**G221S**	-7.123 (-7.462 to -6.764)	-7.232 (-7.419 to -7.038)	0.5538
**A238T**	-7.392 (-7.710 to -7.053)	-7.232 (-7.419 to -7.038)	0.3924
**G252A**	-7.559 (-7.909 to -7.192)	-7.456 (-7.598 to -7.309)	0.5925
**V281M**	-7.579 (undefined)	-7.363 (-7.474 to -7.249)	0.6644
**E339K**	-6.750 (-7.443 to -5.984)	-7.363 (-7.474 to -7.249)	0.0478
**R376G**	-7.566 (-7.729 to -7.400)	-7.456 (-7.598 to -7.309)	0.3221

E_{max}

	**Variant E_{max} (95% CI)**	**WT E_{max} (95% CI)**	**Significance (*P*)**
**V45L**	73 (65 to 82)	99 (93 to 107)	**<0.0001**
**P108A**	101 (92 to 112)	102 (98 to 107)	0.8726
**V172A**	80 (73 to 89)	99 (93 to 107)	**0.0007**
**L206V**	134 (123 to 146)	102 (96 to 108)	**<0.0001**
**A218T**	79 (71 to 88)	102 (98 to 107)	**<0.0001**
**G221S**	88 (77 to 100)	99 (93 to 107)	0.0751
**A238T**	94 (83 to 107)	99 (93 to 107)	0.4363
**G252A**	112 (97 to 127)	102 (96 to 108)	0.2147
**V281M**	9 (4 to 18)	102 (98 to 107)	0.0174
**E339K**	26 (18 to 38)	102 (98 to 107)	**0.0006**
**R376G**	128 (121 to 137)	102 (96 to 108)	**<0.0001**

Results shown are point estimates and 95% confidence intervals from dose-response curves generated from three replicate experiments. Variant point estimates are shown next to point estimate from the WT control on the same plate. Statistically significant changes in log(EC50) or E_{max} are shown in bold [(extra sum of squares F test, $P<0.0045$ ($\alpha=0.05$ with Bonferroni correction for 11 comparisons)].

Table S3: Oxytocin-induced β-arrestin-2 recruitment for wild type (WT) and variant OXTRs

Log(EC50)

	**Variant log(EC50) (95% CI)**	**WT log(EC50) (95% CI)**	**Significance (*P*)**
**V45L**	-7.041 (-7.218 to -6.862)	-7.504 (-7.625 to -7.379)	**<0.0001**
**P108A**	-6.893 (-7.048 to -6.735)	-7.295 (-7.485 to -7.099)	**0.0017**
**V172A**	-7.206 (-7.378 to -7.030)	-7.504 (-7.625 to -7.379)	0.0050
**L206V**	-7.533 (-7.880 to -7.167)	-7.655 (-7.850 to -7.457)	0.5756
**A218T**	-7.349 (-7.538 to -7.153)	-7.295 (-7.485 to -7.099)	0.6795
**G221S**	-7.352 (-7.588 to -7.103)	-7.504 (-7.625 to -7.379)	0.2376
**A238T**	-7.392 (-7.603 to -7.169)	-7.504 (-7.625 to -7.379)	0.3463
**G252A**	-7.601 (-7.829 to -7.364)	-7.655 (-7.850 to -7.457)	0.7065
**V281M**	-7.287 (-7.612 to -6.940)	-7.295 (-7.485 to -7.099)	0.9767
**E339K**	-7.074 (-7.287 to -6.857)	-7.295 (-7.485 to -7.099)	0.1975
**R376G**	-7.570 (-7.740 to -7.395)	-7.655 (-7.850 to -7.457)	0.4945

E_{max}

	**Variant E_{max} (95% CI)**	**WT E_{max} (95% CI)**	**Significance (*P*)**
**V45L**	87 (81 to 94)	98 (94 to 103)	0.0056
**P108A**	120 (111 to 129)	103 (95 to 111)	**0.0039**
**V172A**	93 (87 to 99)	98 (94 to 103)	0.1178
**L206V**	149 (131 to 169)	103 (95 to 110)	**<0.0001**
**A218T**	94 (87 to 101)	103 (95 to 111)	0.0942
**G221S**	100 (91 to 109)	98 (94 to 103)	0.7994
**A238T**	105 (97 to 113)	98 (94 to 103)	0.1488
**G252A**	104 (95 to 113)	103 (95 to 110)	0.8088
**V281M**	26 (22 to 30)	103 (95 to 111)	**<0.0001**
**E339K**	51 (46 to 56)	103 (95 to 111)	**<0.0001**
**R376G**	113 (105 to 120)	103 (95 to 110)	0.0478

Results shown are point estimates and 95% confidence intervals from dose-response curves generated from three replicate experiments. Variant point estimates are shown next to point estimate from the WT control on the same plate. Statistically significant changes in log(EC50) or E_{max} are shown in bold [(extra sum of squares F test, $P<0.0045$ ($\alpha=0.05$ with Bonferroni correction for 11 comparisons)].

Table S4: Log(IC50)s for desensitization and internalization curves for wild type (WT) and variant OXTR.

Desensitization

	Variant log(IC50) (95% CI)	WT log(IC50) (95% CI)	Significance (*P*)
**V45L**	-7.781 (-7.969 to -7.541)	-8.314 (-8.474 to -8.146)	**0.0001**
**P108A**	-7.840 (-8.054 to -7.530)	-8.414 (-8.490 to -8.336)	**<0.0001**
**L206V**	-8.414 (-8.609 to -8.200)	-8.427 (-8.541 to -8.310)	0.9040
**V281M**	-8.532 (-8.734 to -8.328)	-8.338 (-8.683 to -8.002)	0.3124
**E339K**	-7.828 (-7.976 to -7.654)	-8.355 (-8.518 to -8.180)	**<0.0001**

Internalization

	Variant log(IC50) (95% CI)	WT log(IC50) (95% CI)	Significance (*P*)
**V45L**	-8.016 (-8.285 to -7.746)	-8.436 (-8.606 to -8.269)	**0.0098**
**P108A**	-7.965 (-8.199 to -7.732)	-8.559 (-8.756 to -8.356)	**0.0003**
**L206V**	-8.556 (-8.670 to -8.441)	-8.657 (-8.909 to -8.384)	0.4626
**V281M**	-8.571 (-9.381 to -7.769)	-8.704 (-9.169 to -8.229)	0.7595
**E339K**	-8.350 (-8.687 to -8.026)	-8.585 (-8.796 to -8.369)	0.2135

Results shown are point estimates and 95% confidence intervals (CI) from dose-response curves generated from three replicate experiments. Variant parameters are shown next to parameters from the WT control from the same experiment. *P* value shown from extra sum-of-squares F test comparing log(IC50) values between variant and WT.

Figure S1: Atoms included in DiffNets analysis.

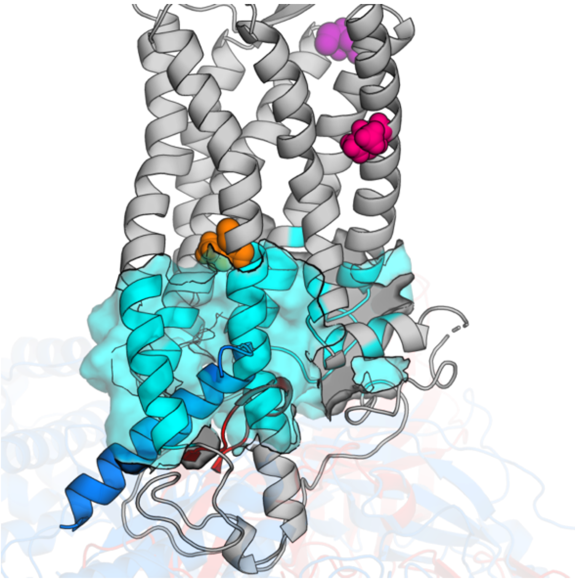

Atoms included in DiffNets analysis (cyan). OXTR homology model (grey) showing variants V45L (magenta), P108A (purple), and V281M (orange). Superimposed structures show β-arrestin-1 (red) and G protein (blue).

Figure S2: Comparison of equilibrium properties calculated from simulations using different clustering methods.

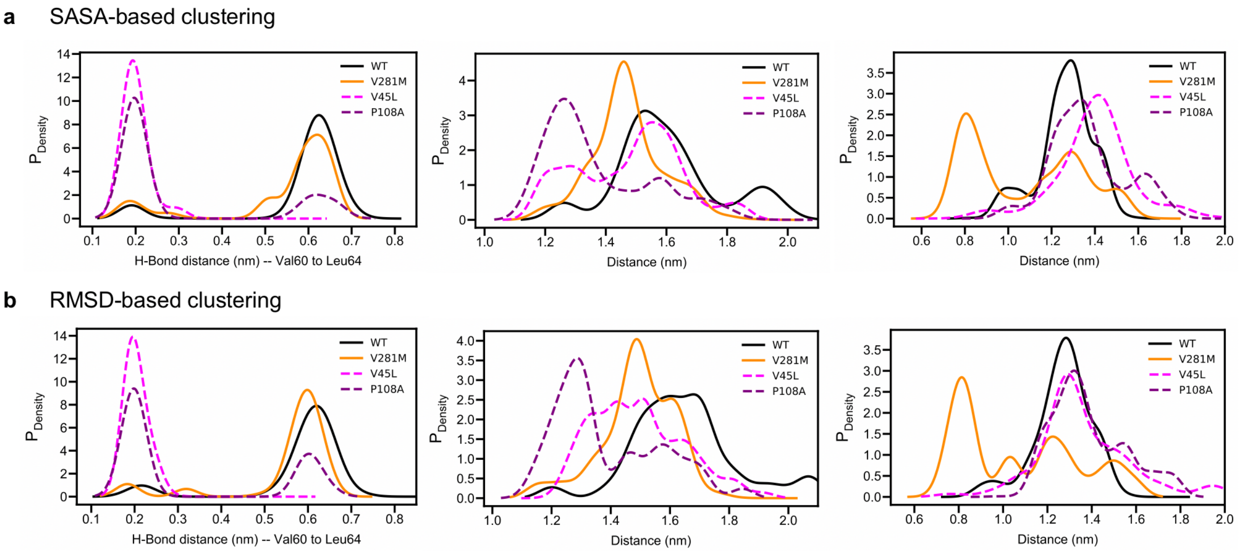

The three distance distributions from Figure 6A, 6C, and 7A are replotted here from left to right using SASA-based clustering (a) and RMSD-based clustering (b). (a) and (b) are highly consistent suggesting that the choice of clustering used prior to MSM construction does not strongly affect the computed equilibrium properties of the system.

Figure S3: Oxytocin-induced ß-arrestin recruitment to wild type (WT) and variant OXTRs.

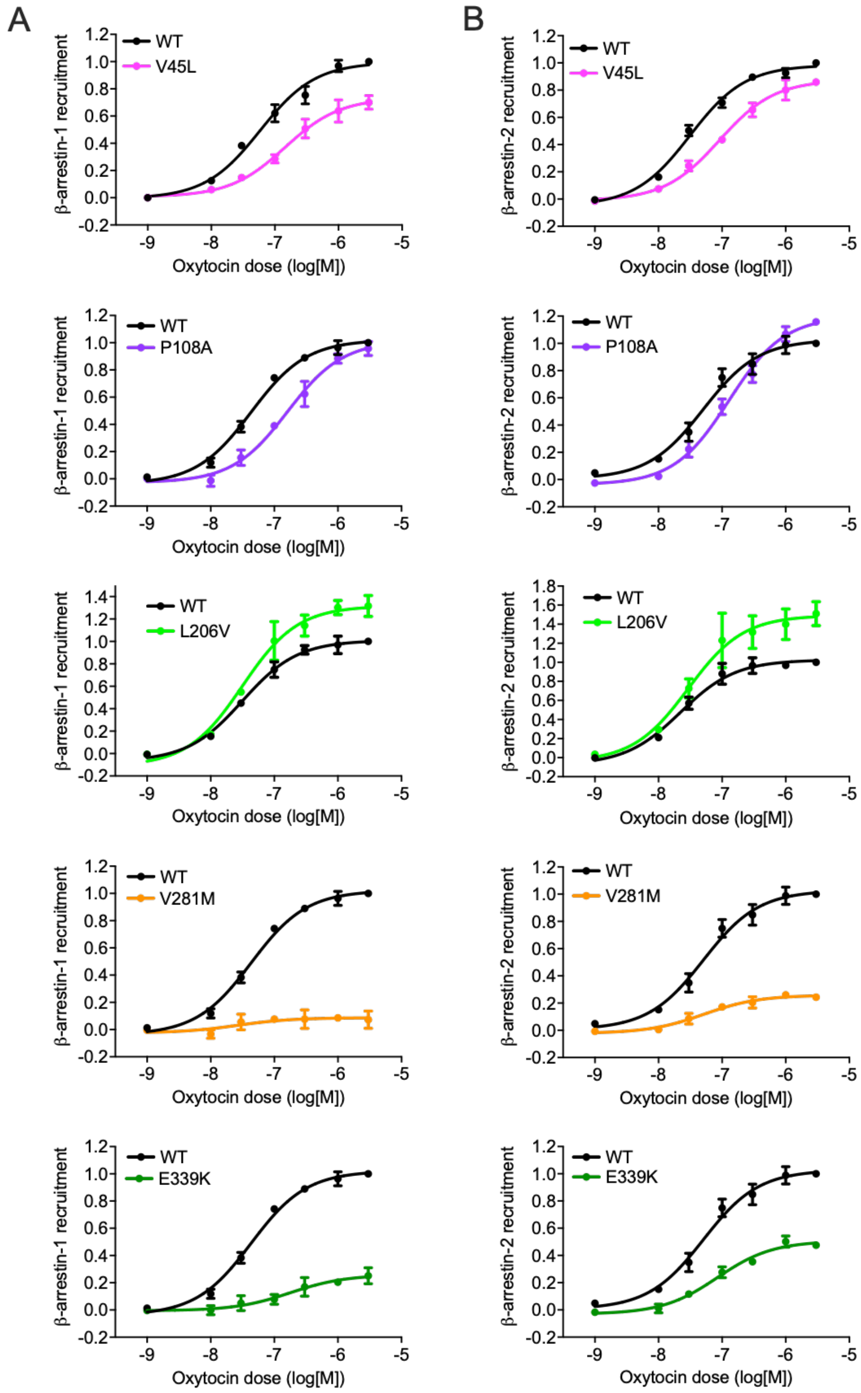

Dose response curves for β-arrestin-1 (A) and β-arrestin-2 (B) recruitment are shown for WT and variant OXTR. Error bars show standard error from *N*=3 independent experiments.

Figure S4: Bias plots for wild type (WT) and variant OXTRs

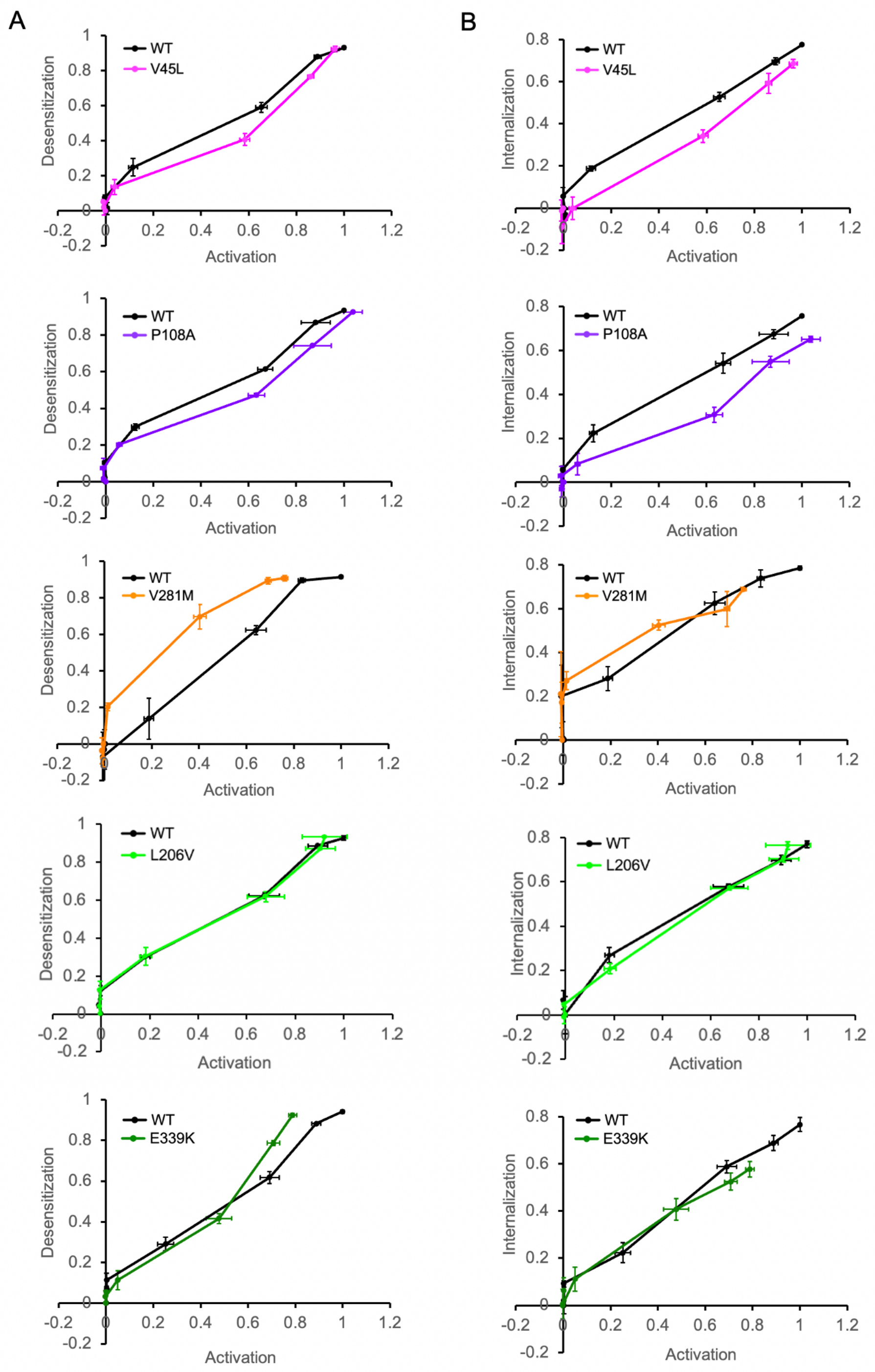

Each point represents activation and desensitization (A) or internalization (B) for one oxytocin dose (10^{-12}-10^{-6} M). Error bars are SEM from $N=3$ independent experiments.
